# Comparison of standard versus accelerated corneal collagen cross-linking for keratoconus: 5-year outcomes from the Save Sight Keratoconus Registry

**DOI:** 10.1038/s41433-023-02641-6

**Published:** 2023-06-27

**Authors:** Himal Kandel, Marco Abbondanza, Aanchal Gupta, Richard Mills, Adam S. Watson, Constantinos Petsoglou, Yves Kerdraon, Stephanie L. Watson

**Affiliations:** 1 The University of Sydney, Faculty of Medicine and Health, Save Sight Institute, Sydney, NSW Australia; 2 Abbondanza Eye Centres, Rome and Milan, Rome, Italy; 3 Adelaide Eye & Laser Centre, Adelaide, SA Australia; 4 South Australian Institute of Ophthalmology, Adelaide, SA Australia; 5 Flinders Medical Centre, Flinders University, Adelaide, SA Australia; 6 Eye Institute, Auckland, New Zealand

**Keywords:** Outcomes research, Corneal diseases

## Abstract

**Objective:**

To compare long-term effectiveness of Standard (UV intensity: 3 mW/cm^2^, duration: 30 min) vs Accelerated (UV intensity: 9 mW/cm^2^, duration: 10 min) corneal cross-linking (CXL) for stabilising keratoconus.

**Methods:**

Data for this observational study were captured through a web-based registry system from the routine clinical practice (15 sites across Australia, New Zealand and Italy). The outcomes were compared using mixed-effects regression models. A total of 100 eyes (75 patients) who had standard CXL and 76 eyes (66 patients) who had accelerated CXL, with a follow-up visit at five-year post-CXL were included.

**Results:**

Both CXL protocols were effective and safe in stabilising keratoconus and improving outcomes. The adjusted mean changes (95% CI) in outcomes were better in standard CXL than in accelerated CXL [visual acuity gain, 10.2 (7.9–12.5) vs 4.9 (1.6–8.2) logMAR letters; pinhole visual acuity 5.7 (3.5–7.8) vs 0.2 (−2.2 to 2.5) logMAR letters; Kmax −1.8 (−4.3 to 0.6) vs 1.2 (−1.5 to 3.9)D; K2 −0.9 (–2.2 to 0.3) vs 0.1 (−1.3 to 1.6)D; MCT –3.0 (−13.7 to 7.7) vs −11.8 (−23.9 to 0.4) µm (*p* values for visual acuity, pinhole visual acuity, Kmax: <0.05; for K2 and MCT: >0.05)]. The frequency of adverse events at the 5-year follow-up visit was low in both groups [standard, 5 (5%; haze 3; scarring 1, epithelial defect 1) and accelerated 3 (3.9%; haze 2, scarring 1)].

**Conclusions:**

Both standard and accelerated CXL were safe and effective procedures for stabilising keratoconus in the long term. The standard CXL resulted in greater improvements in visual acuity and keratometry.

## Introduction

Keratoconus is a progressive and chronic corneal ectatic disorder which typically has an onset in the second decade of life [1, 2]. It results in poor quality vision and reduced quality of life [3–5]. The keratoconic cornea is biomechanically weak which may be the reason for thinning and progression [6]. The introduction of corneal cross-linking (CXL) [7, 8] to halt, slow, or prevent keratoconus progression has revolutionized the treatment of keratoconus by avoiding corneal transplantation in most cases and improving quality of life [9, 10]. Corneal cross-linking has been a standard procedure although evidence on long-term outcomes is rare as there is no other treatment available to stop keratoconus progression.

Corneal cross-linking enhances the biomechanical strength of the cornea [11]. The photochemical reaction between Ultraviolet-A (UVA) and riboflavin (vitamin B2, a photosensitiser) induces bonds in the corneal stroma. In the standard CXL protocol (sCXL), also known as the Dresden protocol, 370 nm UVA wavelength at 3 mW/cm2 intensity is exposed to corneal stroma for 30 min producing a total energy dose of 5.4 J/cm^2^ with riboflavin as the sensitiser [8].

Currently, several variations on sCXL are in clinical practice including the accelerated cross-linking protocols (aCXL) to increase convenience and reduce risks. The aCXL protocols were developed based on the photochemical rule of reciprocity which states that the same reaction may be achieved by decreasing the UV exposure duration and increasing the UV intensity if the cumulative energy dose remains the same [12]. However, differences in outcomes of different protocols (e.g. shallower demarcation line with aCXL than with sCXL [13, 14]) have been reported as the biochemical change may not be the same as photochemical change in the cornea [13–15].

Literature comparing the outcomes of sCXL and aCXL is divided [16–19]. We recently published a large, multinational, real-world, registry study comparing 1-year outcomes of sCXL and aCXL (UV intensity 9 mW/cm^2^ for 10 min), the two most common CXL protocols in the Save Sight Keratoconus Registry (SSKR) [20]. We found that both protocols were similarly safe and effective in stabilising keratoconus. Studies on long-term (≥5 years) outcomes of CXL are rare and are limited further due to the low sample size [21–23]. The evidence on the long-term outcomes of aCXL is particularly scarce. To our knowledge, no study has compared >2 years outcomes of the sCXL and aCXL. This study aimed to compare the long-term efficacy and safety of sCXL vs aCXL (UV intensity 9 mW/cm^2^ for 10 min) for the treatment of keratoconus.

## Methods

The study utilized the data from the SSKR, a web-based, multinational patient database for tracking natural history and treatment outcomes in keratoconus [20, 24]. Patients from 15 sites were included in the current study. As previously described [20], the SSKR collected information on patient demographics, ocular and systemic history, equipment, treatment details, and outcomes. The choice of diagnostic and treatment equipment and the cross-linking protocol were made by the clinicians reflecting real-world clinical practice. The study was performed in accordance with the tenets of the Declaration of Helsinki. Ethics approvals in Australia were obtained from the Sydney Local Health District Ethics Review Committee (RPAH Zone) for the public and the ethics committee of the Royal Australian and New Zealand College of Ophthalmologists for the private sites.

### Inclusion criteria

The study included registry participants diagnosed with keratoconus who had undertaken epithelium-off CXL before December 2016 and had a follow-up visit at 5 years post-CXL. The patients underwent sCXL (UVA intensity 3 mW/cm^2^ for 30 min) or aCXL (UVA intensity 9 mW/cm^2^ for 10 min), both with a cumulative energy dose of 5.4 J/cm^2^. The patients > 50 years old at the time of CXL were excluded. Similarly, the patients with pre-existing ocular conditions (post-LASIK ectasia, corneal dystrophy including Fuch’s endothelial dystrophy, pellucid marginal degeneration, keratoglobus, forme fruste keratoconus, pterygium, cataract, pseudophakia, aphakia, herpes simplex keratitis, herpes zoster keratitis, glaucoma and other optic neuropathy, other neurological disorders, amblyopia, and retinal diseases) were excluded. Patients who had undergone ocular surgery that could impact visual, keratometry or pachymetry outcomes (corneal graft, corneal inlay, intrastromal rings, and refractive surgery including phakic IOL) were excluded. The eyes that had undergone repeated CXL (2 eyes with repeated aCXL) were also excluded.

### Outcome measures

The primary outcome measures included visual acuity, maximum keratometry (Kmax) and central steepest keratometry (K2). Habitual visual acuity was defined as the visual acuity with the available optical correction, if any, during the time of clinical visit reflecting real-world practice [25]. The change in habitual visual acuity was calculated when the correction method (unaided, spectacles or contact lenses) during the follow-up visit was the same as that at the baseline visit [20]. Baseline visit was defined as the clinical visit immediately before the CXL procedure. The secondary outcome measures included minimum corneal thickness (MCT), and the frequency of adverse events within and at 5 years follow-up visit.

### Statistical analysis

The statistical methods were similar to the paper published earlier [20]. The analyses were conducted using the R software (Version 4.1.1; The R Foundation for Statistical Computing, Vienna, Austria). The descriptive statistics included mean, median, percentages, standard deviation (SD), and 95% confidence intervals (CI). The statistical tests for comparing baseline characteristics and crude (unadjusted) changes in outcomes included the t-test (student and paired), chi-square, one-way analysis of variance (ANOVA), Tukey (post-hoc), Wilcoxon rank sum test, and Kruskal Wallis tests where appropriate. A *p* value < 0.05 was regarded as statistically significant. Locally weighted scatterplot smoothing (LOESS) curves were used to visually examine the trends in changes in outcomes.

Mixed-effects regression models were used to determine the adjusted changes in visual acuity, keratometry and MCT with sCXL or aCXL as the main predictor variable (the lme4 package, version 1.1-21) [26]. The models included variables on fixed (age, sex, baseline visual acuity, keratometry, pachymetry) and random (practice and patient) effects [20].

## Results

### General characteristics of the study population

A total of 176 eyes of 141 patients (mean age at the time of CXL, 24.2 ± 7.7 years; 34.0% female) in the SSKR with a baseline visit immediately before CXL and a follow-up visit at 5-year post-CXL were included in the current study. A majority of the patients (70.2%) were from Australia. Spectacles were the habitual optical correction method for most (74.4%). Similarly, Pentacam (Oculus, Wetzlar, Germany) was the most used (86.4%) topographer. Most of the CXL procedures (89.8%) were performed with the Innocross system (IROC Innocross AG, Zug, Switzerland). A bandage contact lens was used after slightly more than half (51.7%) of the CXL procedures (Table 1). Each clinician performed only one type of the CXL protocol.

**Table 1:** Baseline characteristics of the patients and the diagnostic and therapeutic techniques used in their management.

	Standard CXL	Accelerated CXL	*p* value*
Patients (Eyes)	75 (100)	66 (76)	
Age at CXL, mean (SD, min, max) years	24.3 (7.3, 12, 46)	23.7 (7.7, 9, 49)	0.567
Female, *n* (%)	25 (33.3)	23 (34.8)	0.990
Country of residence, *n* (%)			
Australia	36 (48.0)	63 (95.5)	<0.001*
Other	39 (52.0)	3 (4.5)	<0.001*
Ethnicity, *n* (%)			
White	68 (90.7)	38 (57.6)	<0.001*
Other	4 (5.3)	5 (7.6)	0.734
Unspecified	3 (4.0)	23 (34.8)	<0.001*
Optical correction method, *n* (%)			
Spectacles	89 (89.0)	42 (55.3)	<0.001*
Contact lenses	2 (2.0)	5 (6.6)	0.242
Unaided	9 (9.0)	29 (38.2)	<0.001*
Keratometer, *n* (%)			
OCULUS pentacam	79 (79.0)	73 (96.1)	<0.001*
Baush & Lomb Orbscan	7 (7.0)	3 (3.9)	0.518
Others/missing data	14 (14.0)	–	
Crosslinking instrument, *n* (%)			
IROC Innocross	87 (87.0)	71 (93.4)	0.253
Others / missing data	13 (13.0)	5 (6.6)	0.253
Epithelial debridement technique, *n* (%)			
Manual	85 (85.0)	8 (10.5)	<0.001*
Alcohol	15 (13.0)	66 (86.8)	<0.001*
Laser	–	1 (1.3)	-
Adjunct therapies, *n* (%)			
Bandage contact lens after CXL	38 (38.0)	53 (69.7)	<0.001*
Sterile water	2 (2.0)	3 (1.7%)	0.212
Normal saline	–	1 (1.3%)	
No adjunct therapy	62 (62.0)	23 (30.3)	<0.001*

*Statistically significant difference between the Standard and Accelerated CXL groups on t-test, chi-squared or Fisher test.

*CXL* cross-linking.

A total of 100 eyes of 75 patients had sCXL and 76 eyes of 66 patients had aCXL. At baseline, the visual acuity, Kmax, and MCT values were better for the sCXL than for the aCXL (all *p* < 0.05) suggesting the aCXL group consisted of more severe cases at baseline (Table 2).

**Table 2:** Primary outcomes of standard and accelerated corneal cross-linking for keratoconus.

	Overall	Standard	Accelerated	*p* value^a^
Habitual visual acuity (logMAR letters)				
Baseline, mean (SD)	63.5 (18.3)	69.8 (15.1)	55.3 (19.1)	<0.001*
Gain in crude mean (95% CI)	8.4 (6.2–10.5)	8.3 (5.8–10.7)	8.6 (4.2–12.9)	0.899
Gain in adjusted mean (95% CI)		10.2 (7.9–12.5)	4.9 (1.6–8.2)	0.013*
≥70 letters, Baseline/Final (%)	49.4/72	65.0 / 88.2	28.9/42.6	<0.001*/<0.001*
≤35 letters, Baseline/Final (%)	11.4/6.1	4.0/0	21.1/17.0	0.010*/<0.001*
Gain ≥ 10 letters, %	39.4	42.4	34.0	0.454
Loss ≥ 10 letters, %	4.5	2.4	8.5	0.234
Pinhole visual acuity (logMAR letters)				
Baseline, mean (SD)	74.1 (13.0)	78 (13.4)	69 (10.6)	<0.001*
Gain in crude mean (95% CI)	3.8 (2.2–5.4)	4.2 (2–6.4)	3.3 (0.8–5.8)	0.593
Change in adjusted mean (95% CI)		5.7 (3.5–7.8)	0.2 (−2.2 to 2.5)	<0.001*
Kmax (Dioptre)				
Baseline keratoconus severity:				
Kmax <48, %	10.1	11.1	8.6	0.665
Kmax ≥ 48 & ≤ 55, %	32.5	44.4	15.7	<0.001*
Kmax >55, %	57.4	44.4	75.7	0.001*
Baseline, mean (SD)	56.6 (7.0)	54.8 (6.5)	59.1 (7.0)	<0.001*
Final Kmax, mean (SD)	55.6 (7.8)	53.2 (6)	58.8 (8.9)	<0.001*
Change in crude mean (95% CI)	−1 (−1.7 to −0.3)	−1.5 (−2.1 to −0.9)	−0.3 (−1.7 to 1)	0.125
Change in adjusted mean (95% CI)		−1.8 (−4.3 to 0.6)	1.2 (−1.5 to 3.9)	0.030*
Increase ≥ 1D, %	18.1	12.2	26.5	0.033*
Flattening ≥ 1D, %	55.4	64.3	42.6	0.009*
K2 (Dioptre)				
Baseline, mean (SD)	50.6 (6.2)	50.0 (6.5)	51.4 (5.8)	0.118
Final, mean (SD)	50.0 (5.1)	49 (4.3)	51.4 (5.8)	0.004^*^
Change in crude mean (95% CI)	−0.6 (−1.2 to 0)	−0.9 (−1.9 to 0)	−0.1 (−0.7 to 0.5)	0.153
Change in adjusted mean (95% CI)		−0.9 (−2.2 to 0.3)	0.1 (−1.3 to 1.6)	0.233
Increase ≥ 1D, %	13.9	13.1	14.9	0.917
Flattening ≥ 1D, %	35.8	41.4	28.4	0.108

^a^*p* value calculated for comparison between standard and accelerated CXL.

*Statistically significant.

### Primary outcomes

#### Visual acuity

Both protocols improved mean visual acuity (habitual and pinhole) outcomes at a 5-year follow-up visit compared to the baseline visit (all *p* < 0.05; Table 2). Overall, there was a gain of ≥10 logMAR letters in 39.4% patients, gain or loss of <10 logMAR letters in 56.1%, and loss of ≥ 10 logMAR letters in 4.5% eyes.

**Fig. 1:**
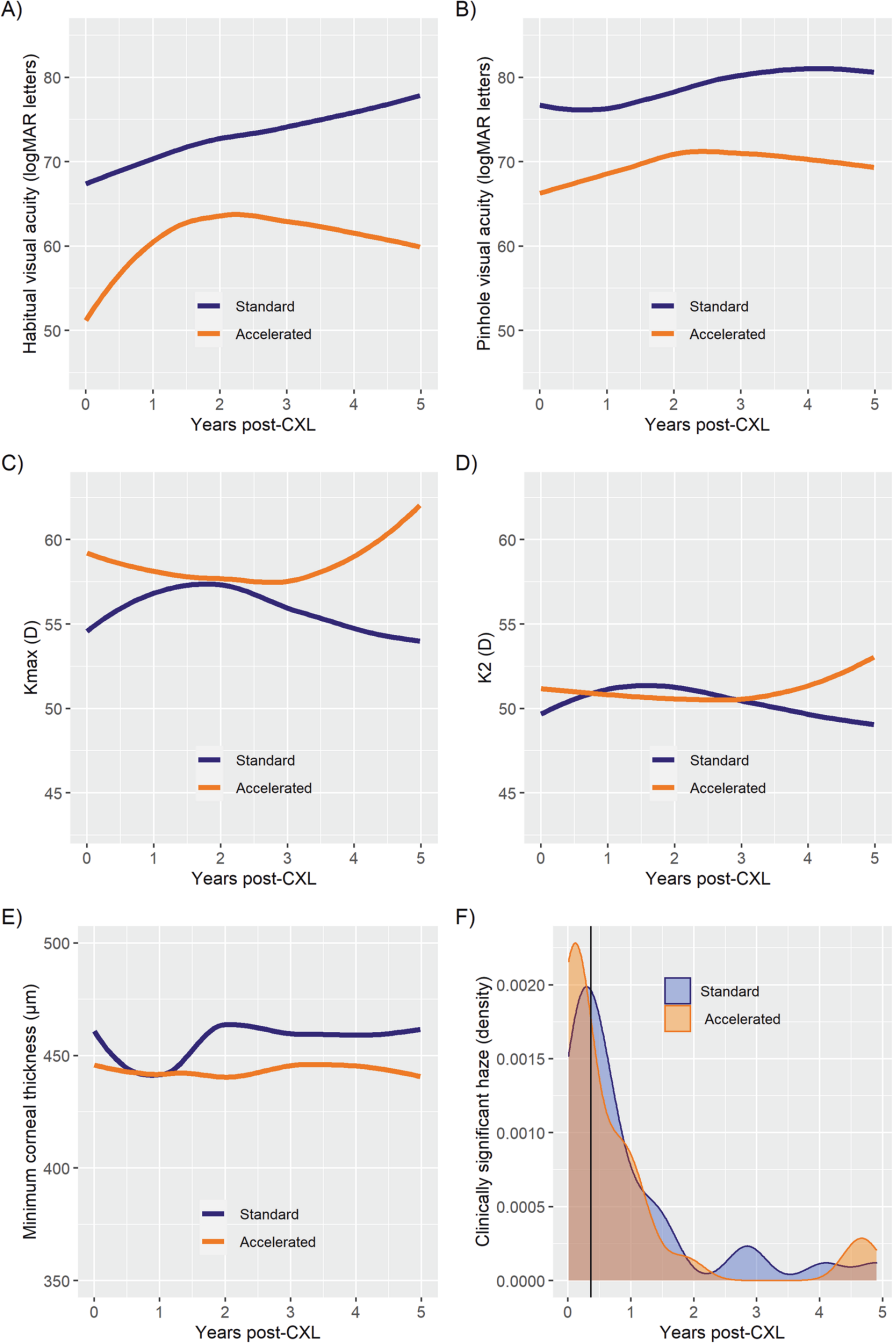
Comparison of standard vs accelerated crosslinking. **A**–**E**: Locally Estimated Scatterplot Smoothing (LOESS) regression line fitted to the data (**A** Habitual visual acuity **B** Pinhole visual acuity **C** Kmax **D** K2 **E** Minimum corneal thickness); **F** Density plot showing clinician-reported clinically significant haze (vertical line represents the median time when haze occurred) Note: A total of 1176 data points (sCXL 443, aCXL 733) were used to plot the figures. For the LOESS curves, the smoothing factor (span) was 0.85. CXL Cross-linking.

The crude (unadjusted) mean gain in visual acuity was similar between the sCXL and aCXL groups (8.3 vs 8.6 logMAR letters; *p* = 0.899). However, the sCXL group had a higher adjusted mean gain in visual acuity (10.2 vs 4.9 logMAR letters; *p* = 0.013; Fig. 1A). Similarly, the proportion of eyes with gain (sCXL, 42.4% vs aCXL, 34.0%) or loss (sCXL, 2.4% vs aCXL, 8.5%) of habitual visual acuity ≥ 10 letters were more favourable for the sCXL. However, the differences in the proportions were not statistically significant (*p* = 0.454 and 0.234, respectively).

Likewise, the crude mean gain in pinhole visual acuity was similar in the CXL groups (sCXL, 4.2 vs aCXL,3.3 logMAR letters; *p* = 0.593). However, the gain in adjusted mean pinhole visual acuity was higher in the sCXL group (5.7 vs 0.2 logMAR letters; *p* < 0.001; Fig. 1B).

#### Keratometry

The sCXL protocol resulted in a statistically significant decrease in Kmax and K2 (both *p* < 0.05), however, the differences between baseline and follow-up keratometry (Kmax and K2) values for the aCXL were not statistically significant (both *p* > 0.05, Table 2).

The sCXL group had a larger reduction in crude and adjusted Kmax (Table 2). The difference in crude mean change in Kmax between the groups was not statistically significant (sCXl −1.5 D vs aCXL −0.3 D, *p* = 0.125). Whereas, the differences in adjusted mean changes in Kmax was statistically significant (sCXL -1.8 vs aCXL 1.2, *p* = 0.030). The proportion of patients with a decrease of ≥1D Kmax was also higher in the sCXL group (sCXL 64.3% vs aCXL 42.6%, *p* = 0.009, Table 2). Considering ≥1D increase in Kmax as the criteria for progression [20], 12.2% eyes with sCXL and 26.5% eyes with aCXL had progression at five years post-CXL (*p* = 0.033). Among the cases which had ≥1D increase in Kmax, the median change in Kmax (interquartile range) was 1.95 (1.18)D in sCXL and 2.40 (2.77)D in aCXL groups; the difference between the CXL groups was not statistically significant (*p* = 0.189).

Similarly, the sCXL group had a larger reduction in crude (sCXL −0.9 vs aCXL −0.1D) and adjusted K2 (sCXL −0.9 vs aCXL 0.1D, Table 2). However, the differences were not statistically significant (both *p* > 0.05). Similarly, the proportion of patients with a decrease of ≥1D K2 was also higher in the sCXL group (sCXL 41.4% vs aCXL 28.4%) and the difference did not reach statistical significance (*p* = 0.108). Likewise, the difference in the proportion of the eyes in which the K2 increased ≥ 1D was not statistically significant (sCXL 13.1%, aCXL 14.9%, *p* = 0.917).

### Secondary outcomes

#### Pachymetry

The MCT at baseline and follow-up were similar for the sCXL (*p* = 0.149). However, aCXL was associated with statistically significant thinning in the MCT (*p* < 0.001; Table 3).

**Table 3:** Secondary outcomes of standard and accelerated corneal cross-linking.

	Standard	Accelerated	*p* value
Minimum corneal thickness (MCT), µm			
Baseline, mean (SD)	468.8 (47.9)	451.9 (36)	0.008*
Final, mean (SD)	464.1 (54.9)	441.5 (46.8)	0.003*
Change in crude mean (95% CI)	−5.0 (−11.8 to 1.8)	−11.1 (−17.3 to −4.8)	0.193
Change in adjusted mean (95% CI)	−3.0 (−13.7 to 7.7)	−11.8 (−23.9 to 0.4)	0.240
Increase ≥ 2%, %	28.3	23.0	0.540
Decrease ≥ 2%, %	41.4	37.8	0.750
Adverse events within 5 years post-CXL, n events (n eyes, % eyes)			
Total^a^	47 (17, 17.0%)	36 (17, 22.4%)	0.483
Clinically significant haze^a^	31 (13, 13.0%)	26 (16, 21.1%)	0.222
Scarring^a^	7 (3, 13.0%)	9 (3, 3.9%)	0.400
Persistent epithelial defect	1 (1, 1.0%)	1 (1, 1.3%)	
Sterile infiltrates	2 (2, 2.0%)		
Stromal oedema	1 (1, 1.0%)		
Microbial keratitis	4 (1, 1.0%)		
Recurrent corneal erosion	2 (1, 1.0%)		

*CXL* cross-linking.

^a^*p* value represents statistical significance for difference in ‘number of eyes’ with the occurrence of an adverse event between the standard and accelerated CXL protocols.

*Statistically significant.

The crude mean reduction in MCT at five years was slightly greater in the aCXL group (sCXL 5 µm vs aCXL 11.1 µm), however, the difference was not statistically significant (*p* = 0.193). Similarly, the difference between adjusted mean change in MCT was not statistically significant (sCXL −3.0 µm vs aCXL −11.8 µm, *p* = 0.240). The proportion of eyes with an increase or decrease of MCT by >2% of baseline levels was also similar between the CXL groups (both *p* > 0.05, Table 3).

#### Adverse events

The frequency of adverse events at a 5-year follow-up visit was low in both groups [standard, 5 (5%; haze 3; scarring 1, epithelial defect 1) and accelerated, 3 (3.9%; haze 2, scarring 1)].

Adverse events were reported in 17% of eyes in the sCXL group and 22.4% eyes in the aCXL group (*p* = 0.483; Table 3) within 5 years (at any clinical visit) post-CXL. Haze was the most common adverse event (sCXL, 76.5%; aCXL, 94.1% eyes with adverse events). In the eyes that developed haze, the median number of days when the occurrence of haze was first reported was 41 days post-CXL. It is important to note that haze in most eyes resolved (sCXL, 77% resolved; aCXL, 88% resolved) at the 5 years visit.

The mean gain in visual acuity in the eyes who developed haze within 5 years was similar to those which did not develop haze (haze 8.9 vs no haze 8.2; *p* = 0.870). The mean age of the patients who developed haze was lower than the patients without haze (20.6 vs 25 years; *p* < 0.001). Minimum corneal thickness at baseline was similar (haze, 460.7 vs no haze, 461.7 µm; *p* = 0.908). The eyes with haze had steeper Kmax at baseline than those that did not develop haze (mean Kmax 59.1 vs 56.0 D, *p* = 0.019). The greater Kmax flattening was achieved in the cases with haze (mean; haze, −2.0 vs no haze, −0.8 D; *p* = 0.050). Interestingly, the mean Kmax at follow-up visits between the eyes that developed haze and that didn’t were similar (56.7 vs 55.4 D; *p* = 0.257).

Microbial keratitis was recorded in a 15-year-old Australian male patient which resulted in a corneal scar. He had undergone sCXL in his right eye in 2012 and developed microbial keratitis 6 months post-CXL. From baseline to five years post-CXL, visual acuity (with spectacles) changed from 80 to 60 logMAR letters, Kmax from 63.8 to 55.6D, K2 from 54.3 to 50.8D, and MCT from 459 to 394 µm.

Other adverse events recorded within five years post-sCXL were scarring (*n* = 3), sterile infiltrates (*n* = 2), persistent epithelial defect (*n* = 1), stromal oedema (*n* = 1), and recurrent corneal erosion (*n* = 1). Similarly, scarring was recorded for 3 eyes and persistent epithelial defect was recorded for 1 eye which had had aCXL.

## Discussion

This registry study utilised real-world data from routine clinical practice to evaluate the efficacy and safety of two common CXL protocols five years post-surgery. Both sCXL and aCXL were effective and safe in stabilising keratoconus and improving outcomes. Although the primary purpose of CXL is to stabilise keratoconus, the protocols, especially sCXL, improved visual and keratometry outcomes without causing corneal thinning.

Both protocols improved visual acuity; the gain in mean habitual visual acuity and mean pinhole visual acuity were significantly higher for the sCXL than for aCXL. A greater improvement in visual acuity with sCXL than with aCXL may be associated with greater improvement in keratometry in the former group as the decreased vision in keratoconus is due to the changes in the corneal shape. Previous short-term studies have reported no significant difference in change in visual acuity between sCXL and aCXL groups [15, 20, 27–32]. Given that no previous studies which compare long-term outcomes of sCXL and aCXL are available, we could not conduct a meaningful comparison of the current study findings with the literature. Nevertheless, the mean changes in visual acuity and the proportion of people with visual acuity gain suggested that both CXL procedures were safe and effective.

Previous research has shown that the patients with worse visual acuity and steeper keratometry at baseline are likely to improve more after CXL [33]. In the current study, the visual acuity and keratometry improvements were higher for sCXL despite the aCXL group having the worse mean visual acuity, Kmax and K2 at baseline. The reason for the difference in keratometry outcomes in sCXL and aCXL is unclear. The current study is in agreement with previous short-term research which has suggested that sCXL was more effective in stabilising corneal curvature [27, 30, 34]. Whereas, our previous short-term research [20] and a study by Cinar et al. [28] favoured aCXL, although the differences were not statistically significant. Overall, the current study found a high likelihood of keratoconus being stable or better 5 years after either CXL protocol (stable or improved Kmax in 87.8% eyes with sCXL and 73.5% eyes with aCXL). This is an important finding for educating patients on what to expect after the CXL procedure and provides clinicians with data that may assist in planning the timing of patient follow up visits.

We found that the aCXL protocol caused statistically significant thinning at a 5 years follow-up visit, however, the difference was not clinically meaningful. Although aCXL caused more thinning than the sCXL, the difference in change in MCT between the CXL protocols was not statistically significant. This was similar to our previous short-term research [20]. Other published short-term studies have shown the opposite although the differences were not statistically significant in each case [15, 30–32]. In the current study, the proportion of eyes that had corneal thinning or thickening ≥2% were also similar between the groups. Overall, the current study found that corneal thinning post-CXL may not be of concern in the long term.

The frequency of adverse events within 5 years post-CXL was similar in sCXL and aCXL. Haze was the most frequent adverse event which mostly resolved in the long term. The patients with haze were more likely to be younger and eyes with steeper Kmax at baseline. Our previous study found that most of the haze occurs within three months of the procedure and gradually resolves [20]. This finding was similar in the current study. The CXL procedure involves the desertion of keratocytes. The repopulation of keratocytes may be the main reason for corneal haze within three months post-CXL. Other reasons for short- or long-term haze after CXL could be changes in stromal pressure, glycosaminoglycan hydration, and interactions between proteoglycan and collagen [35]. Overall, at 5-year post-CXL, the frequency of adverse events was low, and the findings demonstrated that CXL is a safe procedure.

The current study showed that the likely need for repeated CXL was low, particularly after sCXL. Two eyes in the aCXL group had repeated CXL whereas no repeated CXL was recorded for sCXL cases. The need for a repeated CXL is a negative outcome suggesting that the first CXL may not have controlled keratoconus progression. A more definite conclusion can be drawn about this as the data in the registry grows to allow a larger-scale study. Indeed, a previous short-term, larger registry study showed that a similar number of eyes needed a repeated CXL in both sCXL and aCXL groups [20].

This study has several strengths. The data reflect routine clinical practice in the treatment of keratoconus utilising commonly used outcome measures such that the findings are directly relevant to the clinical practice. The data also highlights the trends in keratoconus management including the type of topographer, CXL instruments, and so on. The finding that sCXL resulted in better outcomes than aCXL in the long-term is unique and in contrast to short-term studies including an SSKR study which have shown a small or no difference in outcomes of these two protocols, or aCXL resulting in better outcomes [20, 28, 29, 31]. Adequate power of the findings was achieved with a large sample size.

The current study has the inherent limitations of observational studies such as bias due to non-randomisation and confounding. A randomised controlled trial is considered the ‘gold standard’ method to conduct a comparative effectiveness study despite issues with applicability in the clinical practice and generalisability due to strict inclusion criteria [36, 37]. Furthermore, a randomised controlled trial to evaluate long-term outcomes can be costly. The current study findings are particularly useful to inform clinicians of the likely course for their patients post-CXL so that they can counsel patients on what to expect after the CXL procedure in the long term and plan follow-up visits. Nevertheless, it has to be acknowledged that the aCXL group had more participants with severe keratoconus which might have influenced the results. The CXL outcomes may be affected by factors such as age, baseline keratometry, epithelial debridement technique, and eye-rubbing behaviour. The biases due to heterogeneity and confounding were minimised by statistical modelling to control the effects of baseline variables (age, gender, keratometry, visual acuity, pachymetry, practice and patient-related factors). Perhaps selection bias was also not a major issue given that each clinician did only one type of the CXL protocol. Another limitation of the current study was that the reasons for undertaking CXL are not reported as this information was not available in most cases. The indication for cross-linking was determined by the practising clinicians and most of the cases had at least a sign of progression (reduced visual acuity, increased Kmax or K2, and decreased MCT).

In this study, a higher proportion of patients in the aCXL groups used bandage contact lenses post-operatively. The information on the duration of bandage contact lens use, and the use of antibiotics, steroids and/or lubricants were not recorded in the SSKR which is a limitation as these factors may play an important role in establishing a healthy ocular surface. While the role of postoperative management regimes may be crucial in determining short-term CXL outcomes (reducing pain and discomfort, preventing infection, and expediting epithelization), their role in long-term outcomes of CXL is not clear.

In conclusion, sCXL and aCXL were safe and effective procedures for stabilising keratoconus up to five years post-CXL. The sCXL procedure had better visual, keratometry and pachymetry outcomes than the aCXL and was more likely to cause keratoconus regression. Longer-term (>5 years) follow-up studies using the Save Sight Keratoconus Registry will be invaluable. This study findings warrant conducting a randomised controlled trial to compare the protocols in the long term as currently, only short-term (<2 years) trials are available.

## Summary

### What was known before

- Standard corneal cross-linking is relatively a safe and effective procedure to stabilise keratoconus in the short term.
- The variations of standard corneal cross-linking are available that aim to increase convenience and reduce risks.
- There is a paucity of evidence on the long-term outcomes of corneal cross-linking, particularly of the accelerated protocols, in keratoconus.

### What this study adds

- This study found that corneal cross-linking is a safe and effective procedure to stabilise keratoconus in the long term.
- Standard corneal cross-linking protocol resulted in better outcomes than the accelerated protocol.
- The clinicians can be more confident in the long-term safety and efficacy of corneal cross-linking, and at the current level of evidence, the standard corneal cross-linking protocol can be the preferred one as it resulted in greater improvements in visual and keratometry outcomes.

## Data Availability

The datasets generated during and/or analysed during the current study may be available from the corresponding author on reasonable request.
